# Extracellular Vesicles As a Source of Biomarkers for Cancer Diagnosis

**DOI:** 10.32607/actanaturae.27591

**Published:** 2025

**Authors:** L. A. Ovchinnikova, Y. A. Lomakin

**Affiliations:** Shemyakin–Ovchinnikov Institute of Bioorganic Chemistry, Moscow, 117997 Russia

**Keywords:** extracellular vesicles, EVs, oncology, exosomes, liquid biopsy

## Abstract

Extracellular vesicles (EVs) are secreted by nearly all mammalian cells and play a major role in intercellular communication via the transport of various active biomolecules. In cancer, pathological EVs contribute to tumor progression by participating in metastasis, angiogenesis, and immune evasion. Recent advancements in EV research have revealed their potential as noninvasive biomarkers. This review addresses the latest advancements in EV isolation and characterization techniques, elucidates the molecular mechanisms underlying EV biogenesis, and examines their functional roles in cancer progression. Furthermore, we discuss emerging strategies that leverage EV profiling and molecular composition analysis, in conjunction with liquid biopsy technologies, offering possible breakthroughs in early cancer diagnosis and treatment monitoring. By synthesizing these insights, this review emphasizes the growing significance of EVs as versatile and powerful diagnostic tools in oncology.

## INTRODUCTION

Extracellular vesicles (EVs) are spherical lipid bilayer particles that are secreted by all types of cells. EVs are usually classified into exosomes and microvesicles (or ectosomes), based on their origin. However, the diversity of EVs extends beyond this classification. Recent studies have identified many other EV subtypes, such as small ectosomes, apoptotic bodies, migrasomes, large oncosomes, and exophers [1]. In addition, cells can release nonvesicular extracellular nanoparticles, such as supermeres, exomeres, and supramolecular attack particles [2]. To create a unified, standardized classification, the International Society for Extracellular Vesicles (ISEV) has regularly published and updated its MISEV guidelines. These guidelines are an important resource for researchers, because they ensure consistency and accuracy in the characterization of EVs.

Exosomes are the type of EVs that have most widely been studied. They range from 30 to 150 nm in diameter. Exosomes are formed during the release of intraluminal vesicles (ILVs) upon fusion of multivesicular bodies (MVBs) with the plasma membrane, resulting in the secretion of these particles into the extracellular space [3, 4]. While exosomes from normal cells facilitate intercellular communication by transporting various molecules (e.g., proteins, DNA, RNA, lipids), exosomes released by tumor cells are involved in tumor progression, metastasis, angiogenesis, and, in some cases, contribute to chemoresistance [5].

This review analyzes current knowledge about EVs released by tumor cells, the role of EVs in cancer progression, and the potential of EVs as biomarkers.

## ISOLATION AND CHARACTERIZATION OF EXTRACELLULAR VESICLES

Efficient isolation of EVs is an important step in their investigation, but it is often a non-trivial challenge. There are many EV purification techniques, each with its advantages and limitations. However, there is no versatile technique for vesicle isolation; the choice of approach depends on the specific purpose of the research. EVs isolation techniques may be classified as follows: (i) high yield but low purity techniques (polymer precipitation, ultrafiltration); (ii) medium yield and purity techniques (differential ultracentrifugation and size exclusion chromatography); (iii) low yield but high purity techniques (gradient ultracentrifugation, affinity isolation, flow cytometry, and microfluidic approaches) [6]. Often, a combination of these techniques can increase EVs yield and purity [7]. In this case, new techniques for EVs isolation from biological fluids have been under development. One of these approaches, ExoArc, uses a high-throughput inertial microfluidic device that efficiently isolates cell-free plasma for comprehensive RNA and EVs analysis. In conjunction with size exclusion chromatography, this technique affords EVs yields 10-fold higher than those obtained with ultracentrifugation techniques [8].

Various methods are used to characterize EVs. One of the most common approaches is direct visualization of EVs using microscopy; in particular transmission electron microscopy (TEM), scanning electron microscopy (SEM), cryo-electron microscopy (cryo-EM), and atomic force microscopy (AFM). The use of TEM to visualize EVs often results in images of cup-shaped EVs due to sample dehydration, whereas AFM and cryo-EM help preserve the original spherical morphology of EVs, representing their structures more accurately [4]. Another method for characterizing EVs is dynamic light scattering (DLS), which is based on the Brownian motion of dispersed particles. DLS measures the light scattering intensity fluctuations induced by particle motion, which enables one to measure their size distribution. This method is useful for studying the hydrodynamic diameter of EVs and providing information on their size and homogeneity in solution. DLS is widely used for the analysis of EVs in their natural environment [9]. Compared with DLS, nanoparticle trajectory analysis (NTA) enables one to track individual nanoparticles, a tool that is particularly efficient in particle size analysis in complex samples. A significant advantage of NTA is the ability to use fluorescent labels, which allows one to distinguish particles based on their fluorescence signals. Therefore, NTA allows for simultaneous analysis of the sizes of different individual EVs labeled with different fluorescent markers [10]. Although DLS is easier to use and provides faster results, NTA ensures higher accuracy, especially when working with heterogeneous samples. These methods provide insights into the morphology and size of EVs, and investigation of surface molecules is equally important and may help determine the origin of the EVs. Flow cytometry can be used to analyze EV surface markers, but the diameter of EVs is below the detection limit of standard cytometers, and specialized kits are used to overcome these limitations. The mode of action of these kits is based on positive selection using antibodies against EV markers (e.g. CD63, CD81), which are adsorbed on the microparticle’s surface. EVs bound to antibodies remain on microparticles and can be detected by standard cytometers. These kits are able to help more accurately characterize different EV subtypes, based on surface marker expression levels, and to evaluate their functional properties.

## BIOGENESIS AND MOLECULAR COMPOSITION OF EXTRACELLULAR VESICLES

**Fig. 1:**
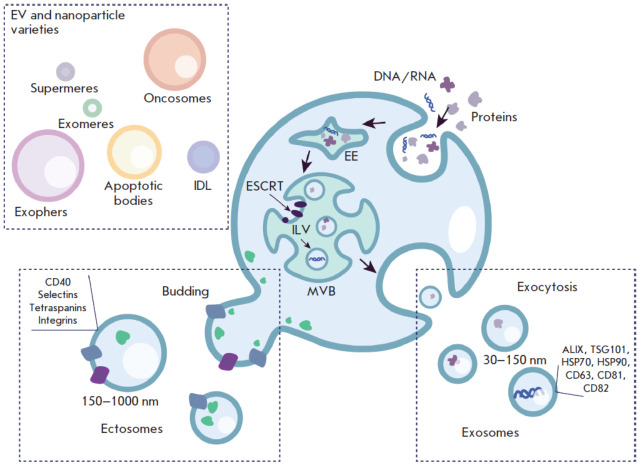
Schematic of exosomes and microvesicles biogenesis. Exosomes form via the endocytic pathway that starts with the invagination of the plasma membrane and formation of early endosomes (EEs). These endosomes mature into multivesicular bodies (MVBs) containing intraluminal vesicles (ILVs). Following fusion of MVBs with the plasma membrane, ILVs are released as exosomes (30–150 nm) into the extracellular space. Microvesicles are formed by direct budding from the plasma membrane, resulting in larger vesicles (150–1,000 nm). IDL – intermediate-density lipoprotein; ESCRT – endosomal sorting complex required for transport

The biogenesis of two main EVs types – exosomes and ectosomes – encompasses various cellular processes (Fig. 1). Exosome biogenesis begins with the formation of early endosomes via invagination of the plasma membrane. These early endosomes can either transport incoming (macro)molecules and supramolecular complexes into intraluminal vesicles (ILVs), which are precursors of exosomes, or transport them back to the plasma membrane. As early endosomes mature, they transform into multivesicular bodies (MVBs) that interact with other organelles, such as the Golgi apparatus, endoplasmic reticulum, mitochondria, and phagosomes. Multivesicular bodies can fuse with the plasma membrane, leading to the secretion of exosomes, or fuse with lysosomes and undergo degradation [11].

There are different pathways of intraluminal vesicles formation within multivesicular bodies. These pathways are divided into ESCRT-dependent and ESCRT-independent ones. Four ESCRT complexes (ESCRT-0, ESCRT-I, ESCRT-II, and ESCRT-III) can interact with the enzymes on the endosomal membrane during exosome biogenesis. The classical ESCRT-dependent pathway involves the recognition of ubiquitinated proteins in the endosomal membrane by ESCRT subcomplexes and VPS4-mediated formation of intraluminal vesicles. An alternative pathway is the syndecan-syntenin-ALIX pathway, where vesicle budding and cargo sorting can occur independently of ESCRT, and VPS4 plays a key role in the final detachment step. The ESCRT-independent pathway uses ceramide, generated from sphingomyelin by nSMase2, that forms lipid raft domains and initiates the maturation of intraluminal vesicles within multivesicular bodies. Thus, the molecular composition of released exosomes depends on the pathways they pass through during their formation. However, there are a number of common proteins typical of the most studied exosomes. These include the proteins involved in membrane transport and fusion (Rab GTPase family and annexins), exosome biogenesis-associated proteins (ESCRT complex proteins, ALIX, TSG101), heat shock proteins (HSP70 and HSP90), tetraspanins (CD63, CD81, and CD82), and cytoskeletal proteins [12]. Besides proteins, characteristic lipids can be found in exosomes. The lipid composition of exosomes depends on the type of producer cells, their developmental stage, and functions. For example, it has been shown that the bis(monoacylglycero) phosphate (BMP) phospholipid stimulates the formation of intraluminal vesicles [13], and that cholesterol is involved in the assembly of the ESCRT system [14]. Sphingomyelin, phospholipids, ganglioside GM3, and cholesterol are the lipids most typical of the exosome membrane [15]. Some exosome membrane lipids may serve as useful diagnostic tools; e.g., phosphatidylserine-exposing exosomes have their origin in malignant cells [16].

Ectosomes (microvesicles), unlike exosomes, bud directly from the plasma membrane of the producer cell (Fig. 1). The molecular mechanisms of ectosome biogenesis are less well understood, but the process is known to involve the ESCRT complex and small GTPase proteins such as ARF1, ARF6, and RhoA. These proteins play an important role in the regulation of cytoskeletal dynamics and membrane remodeling [17]. Furthermore, the inward calcium current and bilayer remodeling play a key role in the formation of ectosomes, influencing their budding from the plasma membrane [18]. Ectosomes carry a wide spectrum of biomolecules, including proteins, lipids, and RNAs, which they transfer to recipient cells, thereby participating in intercellular communication [19]. These EVs rarely possess specific markers, but their association with CD40, selectins, tetraspanins, and integrins has been revealed [20]. In addition, their membranes can incorporate producer cell proteins and lipids [20].

## CONTRIBUTION OF EXTRACELLULAR VESICLES TO CANCER PROGRESSION

EVs are secreted by all types of cells and involved in many pathological processes in the human body, including tumor progression. The tumor microenvironment consists of immune and stromal cells, blood vessels, and the extracellular matrix and plays an active role in tumor progression [21]. The interaction between the tumor microenvironment and cancer cells is partially mediated by EVs [22]. EVs and their contents are able to stimulate tumor growth and progression, cause inflammation, and facilitate tumor escape of immune surveillance [23].

One of the main sources of pathogenic cancer cell-derived EVs are cancer-associated fibroblasts (CAFs), which are important components of the tumor microenvironment in solid tumors. These fibroblasts secrete the cytokines and growth factors that play a key role in tumor growth, angiogenesis, inflammation, and metastasis [24]. CAF-derived exosomes (CDEs) are enriched in bioactive molecules, including numerous signaling factors, nucleic acids, functional proteins, and small metabolites, and they likewise play a significant role in tumor microenvironment modulation via the stimulation of tumor growth, metastasis, and resistance to therapy [25]. CDEs have been shown to inhibit mitochondrial oxidative phosphorylation, alter carbon metabolism, and promote tumor growth [26]. These EVs contain metabolites, in particular amino acids, lipids, and citric acid cycle intermediates, that can be utilized by tumor cells [26]. In addition, these EVs enhance the migratory and invasive capabilities of cancer cell lines, such as SKOV-3 and CAOV-3, and they stimulate epithelial-mesenchymal transition, which is largely a product of elevated TGFβ1 levels [27]. In an animal model of breast cancer (BC), CDEs were shown to enhance tumor cell motility and invasive activity [28]. These exosomes were taken up by tumor cells, providing them with Wnt11, a signaling protein associated with tumor progression. In the case of pancreatic cancer, EVs secreted by tumor-associated fibroblasts increased the chemoresistance-inducing factor (Snail) in recipient epithelial cells and promoted their proliferation and capacity for drug resistance. Inhibition of CDE release reduced the survival of co-cultured epithelial cells, signifying the important role of CDEs in maintaining drug resistance [29].

The pathogenic role of tumor-associated fibroblasts and their EVs is well-documented; however, the molecular mechanisms underlying the reprogramming of normal fibroblasts into tumor-associated ones are poorly understood. One potential mechanism involves the EV-mediated transport of pathogenic microRNAs (miRNAs). A new potential pathway of intercellular communication has been identified in melanoma cells inducing fibroblast transformation via EV-transported miRNAs [30]. It has been shown that melanoma cell-secreted EVs deliver miR-92b-3p into normal fibroblasts, and that the accumulation of this miRNA in the cells correlates with their transformation into tumor-associated fibroblasts [29].

Ascites, which is the accumulation of fluid in the peritoneal cavity, often develops in various pathological conditions, including cancers, and it is another component of the tumor microenvironment, as well as an important source of EVs [31]. In high-grade serous ovarian cancer, ascites fluid was shown to contain EVs originating predominantly from macrophages and fibroblasts rather than tumor cells [32]. A proteomic analysis revealed that ascites-specific EV markers were able to predict patient survival more accurately than traditional cellular markers. EVs derived from ascites (EXOAscites) from gastric cancer patients were also shown to stimulate invasiveness and angiogenesis in a three-dimensional autologous tumor spheroid microfluidic system. EXOAscites delivered the MET oncogene into tumor cells, stimulating oncogenic signals. Modified MET-depleted EVs reduced tumor progression, a sign of potential for targeted therapy [33].

EVs play a significant role in the stimulation of tumor angiogenesis. For example, a known angiogenesis inducer, E-cadherin, is secreted in the form of exosomes [34]. In addition, miR-21, which is present in cancer-associated fibroblast EVs, is delivered into endothelial cells in multiple myeloma, where it regulates angiogenesis [35]. EVs also promote the formation of a pre-metastatic niche, a microenvironment meant for the colonization of circulating tumor cells in specific organs. EVs isolated from pancreatic ductal adenocarcinoma were identified as carriers of the migration inhibitory factor (MIF), a key component in the formation of the pre-metastatic niche in the liver. Blocking MIF in these EVs effectively prevented both pre-metastatic niche formation and subsequent liver metastases. These EVs activated hepatic stellate cells and stimulated extracellular matrix remodeling. This process resulted in the accumulation of fibronectin that recruits macrophages, thereby creating a microenvironment supporting liver metastasis [36].

Another input from EVs in tumor progression is their ability to modulate the immune response. EVs isolated from the cells of chronic lymphocytic leukemia patients induced an immunosuppressive phenotype in monocytes. These EVs stimulated the release of CCL2, CCL4, and interleukin-6 and induced PD-L1 expression via delivery of the non-coding RNA hY4 [37]. PD-L1 was also detected on the surface of glioblastoma-derived exosomes that fostered PD-L1- dependent inhibition of T-cell activation [38]. Tumor EVs were shown to transfer fatty acids to dendritic cells, which led to lipid accumulation and increased fatty acid oxidation, causing dendritic cell immune dysfunction [39].

## EXTRACELLULAR VESICLES AS A TOOL FOR CANCER DIAGNOSIS. LIQUID BIOPSY

**Fig. 2:**
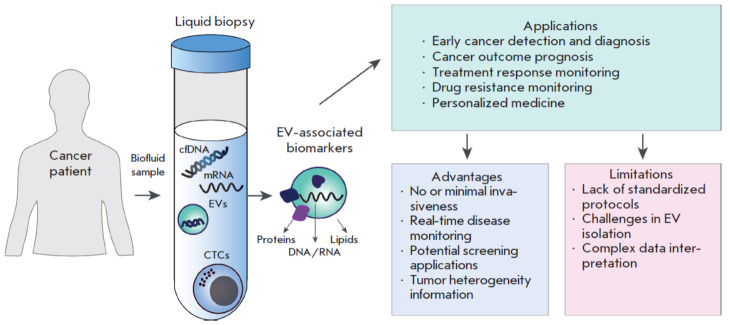
Application of EVs in liquid biopsy for cancer diagnosis. Key elements analyzed by liquid biopsy include circulating cell-free DNA (cfDNA), extracellular vesicles (EVs), mRNAs, circulating tumor cells (CTCs), and tumor-derived metabolites

EVs can be isolated from all types of human biological fluids, in particular blood, tears, urine, saliva, cerebrospinal fluid (CSF), etc. This versatility makes EVs a promising tool for cancer diagnosis, especially in terms of liquid biopsy. Liquid biopsy is an innovative technique used to analyze circulating tumor cells, extracellular nucleic acids, and EVs (Fig. 2). This minimally invasive method enables real-time monitoring of tumor progression [40]. The advantages of EVs analysis using liquid biopsy are as follows: (1) higher EVs concentrations in biological fluids than in circulating tumor cells; (2) EVs, compared with circulating DNA, provide a better insight into producer cells; and (3) the high biological stability of EVs in the aggressive tumor environment [41]. EVs isolated from tumor cells carry a wide range of cytosolic and surface proteins, DNAs, RNAs, as well as various lipids and glycans; so, they can potentially be used in screening for early cancer stages, monitoring cancers, and predicting the response to therapy. Below, we discuss the application of EV analysis to the diagnosis of the most common cancers using liquid biopsy.

**Prostate cancer**

Prostate cancer (PCa) is one of the cancers that has been successfully diagnosed using liquid biopsy. Although the introduction of prostate-specific antigen (PSA) testing has significantly improved diagnostics, there remains a need for biomarkers in order to more accurately track disease progression [42]. In a study using plasma from PCa patients, genomic profiling of EV-associated DNA (EV-DNA) provided tumor characteristics and was in correlation with disease progression, whereas the investigation of EV-associated RNA (EV-RNA) provided insight into the tumor response at early stages of the therapy [43]. Specific miRNAs present in EVs may also be considered as potential biomarkers of PCa. In particular, miR-375, miR-21, and miR-574 were identified in EVs isolated from the serum of PCa patients [44]. In addition, miR-21 and miR-375 were also detected in urinary EVs, indicating that these markers may be used for noninvasive diagnostics [45]. Another EV-associated miRNA, miR-141, was also detected in both the serum and urine of PCa patients, suggesting its potential as a marker for monitoring PCa [46, 47]. It should be noted that PSA was also found in EVs isolated from PCa patients, suggesting that EVs may be used as a source of clinically relevant information [48]. The presence of these specific miRNAs and protein markers in EVs emphasizes their potential role as biomarkers for early detection, progression monitoring, and treatment response assessment in PCa.

**Colorectal cancer**

Colorectal cancer (CRC) is the third most common malignancy worldwide [49]. Traditional diagnostic methods for CRC are invasive and often painful. The development of new, noninvasive diagnostic tools may reduce mortality rates through earlier diagnosis [50]. Most of the EV-associated biomarkers for CRC are RNAs (in particular, miRNAs). A meta-analysis of 159 publications revealed three miRNAs common to all stages of the disease: miR-146a-5p, miR-22-3p, and miR-23b-3p [51]. In addition, seven miRNAs specific to certain CRC stages were identified: stage I – miR-301a-3p and miR-548i; stage IIIA – miR-23a-3p; and stage IV – miR-194-3p, miR-33a-3p, miR-485- 3p, and miR-194-5p [51]. However, the levels of these markers in biological fluids vary significantly, which emphasizes the need for their further validation. Several types of EV-miRNAs have been identified in serum, including let-7a-5p, let-7c-5p, let-7f-5p, let-7d- 3p, miR-423-5p, miR-584-5p, miR-30a-5p, miR-99-5p, miR-150-5p, miR-26-5p, and miR-204-5p [52]. A bioinformatics analysis revealed that the let-7 miRNA family targets the key genes in the TGF-β signaling pathway, in particular TGFβRI and SMAD2, which play significant roles in tumorigenesis. In addition, five more EV-miRNAs (hsa-miR-126, hsa-miR-139, hsa-miR-141, hsa-miR-29c, and hsa-miR-423) displaying high potential as CRC markers have been identified. The miRDIP database was used to establish links between these miRNAs and their target mRNAs involved in the regulation of key pathways, such as the B-cell receptor signaling pathway and glycosphingolipid biosynthesis [53]. Long non-coding RNAs (lncRNAs) can also contribute to CRC progression and serve as prognostic markers of the disease [54, 55]. Not only EV-RNAs, but also some proteins present in EVs can be potential markers of the disease. For example, the prion protein PrPC, found in EVs in CRC, is involved in the formation of conditions for metastasis. This occurs due to increased endothelial permeability and the enhanced secretion of angiogenic factors. A potential new therapeutic approach to control CRC metastasis is chemotherapy combined with anti-PrPC therapy [56].

**Hepatocellular carcinoma**

Hepatocellular carcinoma (HCC) is one of the most common types of primary liver cancer. It’s prognosis, despite advances in treatment, remains unfavorable in most cases. Growing evidence suggests that EVs may serve as specific diagnostic – and even prognostic – biomarkers for HCC [57]. MiRNAs stand out among the most studied exosomal biomarkers for HCC. Some exosomal miRNAs can also be used to choose a treatment strategy at late HCC stages [58]. For example, a panel of miRNAs identified as potential biomarkers includes miRNAs overexpressed in HCC patients: miR-224, miR-21, miR-210-3p, miR-93, miR-92b, miR-155, and miR-665 [59]. In contrast, the expression level of miRNAs, such as miR-718, miR-744, miR- 9-3p, and miR-125b, is decreased in HCC patients. Combining several miRNAs into diagnostic panels may improve diagnostic accuracy. A combination of miR-26a, miR-29c, and miR-199a was shown to effectively discriminate between HCC patients and healthy subjects (AUC = 0.994), as well as between HCC patients and cirrhosis patients (AUC = 0.965) [60]. RNAs carried by EVs, such as circular RNAs (circRNAs), also demonstrate prognostic potential in HCC. For example, the hsa_circ_0029325 level in EVs may be used to predict disease outcome [61]. Another type of EV-derived RNAs that may be used to diagnose HCC is PIWI-interacting RNAs (piRNAs), which are involved in cancer progression. Expression of serum EV-derived piRNAs is elevated in HCC patients, and some of them (e.g., piR-15254, piR-1029, novel-piR-35395, novel-piR-32132, and novel-piR-43597) are potentially usable in HCC diagnosis even in patients with a low tumor burden [62].

EV proteins may also serve as valuable prognostic biomarkers in HCC. For example, decreased CD31 levels in EVs from HCC patients were shown to correlate with HCC recurrence 12 months after surgery [63]. Proteomic profiling yielded a panel of differentially expressed proteins – VWF, LGALS3BP, TGFB1, SERPINC1, HPX, HP, HBA1, FGA, FGG, and FGB – that may form the basis for an HCC diagnostic panel [64]. MiRNAs, circRNAs, piRNAs, and EV proteins are promising noninvasive biomarkers for improving HCC diagnosis, prognosis, and treatment monitoring, and this opens up new opportunities for personalized patient care.

**Pancreatic cancer**

Pancreatic cancer (PC) is the third leading cause of cancer-related deaths [65]. The most common pancreatic cancer is pancreatic ductal adenocarcinoma, which accounts for more than 90% of all PC cases. PC is associated with high mortality; only 10% of patients survive 5 years [66]. Early diagnosis is crucial to improve the prognosis in this disease. Recent advances in machine learning have facilitated the identification of novel potential EV-based biomarkers that may aid in the early diagnosis of PC. Machine learning analysis of EV proteins proposed a panel of seven potential PC biomarkers (mucin-1, sialylated Lewis x antigen, ferritin, fibroblast growth factor 2, human epidermal growth factor 3, leptin, and prolactin, AUC = 0.971) [67]. Another promising PC biomarker, whose concentration is increased in EVs, is glypican-1. Detection of glypican-1 in EVs demonstrated 100% sensitivity and specificity in the diagnosis of all stages of PC, efficiently distinguishing pancreatic cancer patients from healthy subjects or chronic pancreatitis patients (AUC = 1.0) [68]. In addition, miR-21 found in the EVs of PC patients may also be used as a biomarker and prognostic factor of overall survival. Elevated miR-21 levels, in combination with miR-4525 and miR-451a, were shown to exhibit a high potential as biomarkers for the identification of patients with a high recurrence risk and poor prognosis [69]. Elevated miR-191 levels were also detected in a subset of PC patients compared to the controls [70]. Some EV glycans and lipids also appear to have potential as diagnostic tools for PC, emphasizing the significance of diverse EV molecules in the liquid biopsy of this cancer type [71].

**Lung cancer**

Lung cancer (LC), which affects millions annually, remains one of the most frequently diagnosed cancers and the leading cause of cancer-related mortality [72]. Recent advances in multiplexed EV profiling and machine learning have opened up new opportunities for the study of EVs released by lung cancer cells [73]. For example, a system for detecting EV membrane proteins has been developed based on Forster resonance energy transfer. This system was used to identify potential diagnostic markers for early-stage LC (CEA, PD-L1, EpCAM, and CA125) [74]. Another method based on a dielectrophoretic chip revealed elevated miR-21, miR-191, and miR-192 levels in EVs isolated from the blood plasma of lung cancer patients [75]. Additional EV miRNA panels demonstrated their efficiency in the diagnosis of various LC subtypes at early stages. For example, miR-483-3p was proposed as a biomarker for early small cell lung cancer, and miR-152-3p and miR-1277-5p were proposed for early non-small cell lung cancer [76]. In addition, EVs glycan profiling may also be used in the diagnosis of lung cancer. An EV-GLYPH assay, which is based on microfluidic approaches, was used to identify unique glycan signatures of EVs from non-transformed and malignantly transformed lung cells. In a clinical study, that assay successfully differentiated patients with early-stage lung cancer from those with benign nodules [77].

**Breast cancer**

Breast cancer (BC) is the most common cancer in women. In high-income countries, breast cancer is estimated to be diagnosed in every eighth woman by age 85 years [78]. Molecular profiling of the EVs in BC is a powerful toll for early noninvasive diagnosis, prognosis, and disease monitoring [79]. Proteomic profiling of EVs isolated from BC cell lines was shown to differentiate between different BC subtypes more effectively than profiling of the tumor cells themselves [80]. It was also noted that the protein composition of EVs secreted by BC cells largely reflects their molecular subtype (e.g., HER2-positive or triple-negative BC) [80]. In another study, the analysis of EVs from the plasma of BC donors identified 10 candidate biomarkers, whose levels were higher in BC patients than in healthy subjects (CD3, CD56, CD2, CD25, CD9, CD44, CD326, CD133/1, CD142, and CD14). The lipid profile of EVs, in particular sphingolipids and phospholipids, was shown to significantly differ from that of the tumor cells secreting EVs, which were more enriched in triglycerides and fatty acids. EVs isolated from the plasma of BC patients are characterized as sources of lipid biomarkers for the early detection of BC and its subtypes (ER/PR+, HER2+, and triple-negative BC) [81]. In addition, miRNAs obtained from EVs may also be used for BC diagnosis [82].

The main markers mentioned in this review are listed in Table 1.

**Table 1:** EV-associated markers for cancer diagnosis

Biomarker type	Name	Associated cancer	Reference
	piR-15254 ↑ piR-1029 ↑ novel-piR-35395 ↑ novel-piR-32132 ↑ novel-piR-43597 ↑	HCC	[62]
	miR-4525 ↑ miR-451a ↑	PC	[69]
	miR-191 ↑ miR-192 ↑	LC	[75]
	miR-483-3p ↑ miR-152-3p ↑ miR-1277-5p ↑	LC	[76]
	miR-375 ↑	PCa	[44, 45]
	miR-574 ↑	PCa	[44]
Proteins	Cellular prion protein	CRC	[56]
	CD31	HCC	[63]
	Von Willebrand factor Galectin-3-binding protein Transforming growth factor beta 1 Antithrombin III Hemopexin Haptoglobin Hemoglobin subunit alpha 1 Fibrinogen alpha chain Fibrinogen gamma chain Fibrinogen beta chain	HCC	[64]
	Mucin-1 Sialylated Lewis x antigen Ferritin Fibroblast growth factor 2 Epidermal growth factor 3 Leptin Prolactin	PC	[67]
	Glypican-1	PC	[68]
	CEA PD-L1 EpCAM CA125	LC	[74]
	PSA	PCa	[48]
Lipids/ phospholipids	Ceramides Sphingomyelins Hexosylceramides Lysophosphatidylcholines Lysophosphatidylethanolamines Phosphatidylcholines Plasmalogens – phosphatidylethanolamines with an ether bond	BC	[81]

Note. EV – extracellular vesicle;

CRC – colorectal cancer;

PC – pancreatic cancer;

LC – lung cancer;

BC – breast cancer;

HCC – hepatocellular carcinoma;

PCa – prostate cancer;

CA125 – cancer antigen 125;

CEA – carcinoembryonic antigen;

EpCAM – epithelial cell adhesion molecule;

PD-L1 – programmed cell death receptor 1 ligand;

PSA – prostatespecific antigen.

The up (↑) and down (↓) arrows indicate an increase or a decrease, respectively, in the RNA content in extracellular vesicles in samples from cancer patients compared with those from healthy donors.

## INNOVATIVE METHODS FOR IMPROVING EXTRACELLULAR VESICLES DETECTION

An efficient search for EV-based biomarkers requires one to increase the sensitivity of the means used to detect those markers compared with that offered by existing classical methods such as mass spectrometry and Western blotting. The use of artificial intelligence and machine learning methods may significantly improve the detection limit of EV-based biomarkers by liquid biopsy. One of the approaches that improves EV detection is fluorescence polarization using aptamers for the detection of extracellular nanovesicles (FluoPADE) [83]. This method is based on the use of DNA aptamers and fluorescence polarization to detect EVs in human plasma and the culture medium. The specificity of the assay is achieved by fixation of the EVs with antibodies and subsequent detection using a DNA aptamer that targets a specific EV biomarker. This method can be used for early cancer detection, detection of micrometastases, and the monitoring of minimal residual disease. Another approach involves DNA-based barcoding of EVs to explore the protein composition of their surface [84]. One of the advantages of this technology is the ability it affords to investigate the composition of individual exosomes. Also, a method based on nanostructured 3D sensors was developed for the molecular and functional profiling of EVs from cancer stem cells. These highly sensitive sensors were able to detect up to 10 individual EVs in 10 μL, and when combined with artificial intelligence algorithms, allowed one to separate cancer samples from normal ones with 100% sensitivity and 100% specificity [85]. Another method, DNA cascade reaction-triggered individual EV nanoencapsulation (DCR-IEVN), enables the encapsulation of EV subpopulations directly from clinical serum samples. This approach, when integrated with machine learning algorithms, proved highly accurate in diagnostics for HCC [86]. Hoshino et al. performed large-scale proteomic analyses of EVs from various tissues, cells, and biological fluids [87]. They showed that classic EV markers such as CD63, TSG101, flotillins, and ALIX were underrepresented in human plasma EVs. Instead, alternative markers for EV isolation such as MSN, FLNA, STOM, and RAP1B were proposed by the group. Then, machine learning methods were used to identify a panel of EVs proteins specific to certain tumor types. The technique that can be used to classify cancers of unknown primary origin. Proteins and the specific RNAs in individual EVs can be detected using a SPIRFISH technique that combines interferometric reflectance sensor technology with fluorescence in situ hybridization, which ensures high detection sensitivity and specificity [88].

Modern EVs research actively uses artificial intelligence. For example, deep learning algorithms were used in miRNA profiling at the individual EV level [89]. This method combines total internal reflection fluorescence (TIRF) imaging, which simultaneously detects several miRNAs in individual EVs, with an algorithm for automated image analysis. Another deep learning algorithm uses nanoplasmonic spectra to analyze mutated exosomal proteins. The technique may be promising in the efforts to monitor the efficiency of cancer therapy [90].

The limited availability of some biological fluids has prompted researchers to develop innovative methods for EVs isolation. It has been proposed to use cellulose nanosheets that can efficiently capture EVs from a small volume of liquid for subsequent sequencing of small RNAs [91]. Liquid biopsy of EVs offers many advantages compared with classical diagnostic methods. First, it is a noninvasive method that can minimize the need for procedures such as puncture or tissue biopsy, providing patients with more options and helping monitor disease progression and therapy effectiveness. Another of the advantages of this method is the ability it affords one to analyze all biological fluids, which allows for a comprehensive characterization of various tumors.

## CONCLUSIONS

EVs are critically involved in tumor progression. The ability to transport biologically active molecules and alter the tumor’s microenvironment makes EVs potent mediators of tumor progression, metastasis, and immune evasion. Furthermore, EVs are promising tools in the early diagnosis and monitoring of cancers using liquid biopsy techniques. Recent advances in EV isolation and characterization have significantly improved accuracy and efficiency in their investigation, in particular in the field of oncology. The development of innovative methods such as high-throughput microfluidic platforms and machine learning algorithms has increased capabilities in EV detection and analysis and helped to more thoroughly characterize their molecular composition and functional properties. Therefore, investigation of the abnormalities in the molecular composition of EVs in cancers opens up enormous potential for future personalized medicine and tumor diagnosis.

## Abbreviations

EV: extracellular vesicle
EV-DNA: EV-associated DNA
EV-RNA: EV-associated RNA
cfDNA: cell-free DNA
HCC: hepatocellular carcinoma
CRC: colorectal cancer
PC: pancreatic cancer
LC: lung cancer
BC: breast cancer
PCa: prostate cancer
circRNA: circular RNA
CAF: cancer-associated fibroblast
CDE: CAF-derived exosome
EE: early endosome
ESCRT: endosomal sorting complex required for transport
IDL: intermediate-density lipoprotein
ILV: intraluminal vesicle
MVB: multivesicular body
piRNA: PIWI-interacting RNA
PSA: prostate-specific antigen
